# Chemotherapy-Induced Leukoencephalopathy Revealed by Seizure and Alteration of the Mental Status

**DOI:** 10.7759/cureus.39364

**Published:** 2023-05-23

**Authors:** Mohammed El Aissaouy, Badie Douqchi, Ghizlane El Aidouni, Houssam Bkiyar, Brahim Housni

**Affiliations:** 1 Intensive Care Unit, Mohammed VI University Hospital Center, Oujda, MAR; 2 Anesthesiology, Mohammed I University, Oujda, MAR; 3 Intensive Care Unit, Mohammed I University, Oujda, MAR; 4 Anesthesiology/Critical Care Unit, Mohammed VI University Hospital Center, Oujda, MAR; 5 Intensive Care Unit/Anesthesiology, Mohammed I University, Oujda, MAR

**Keywords:** diffusion-weighted magnetic resonance imaging, alterations in mental status, seizure, leukoencephalopathy, 5-fluorouracil

## Abstract

Leukoencephalopathy is progressive demyelination of the white matter, induced by a variety of factors. Among the causes of leukoencephalopathy, chemotherapy is an uncommon cause that generates potentially reversible lesions. The clinical presentation is classically made of alterations in mental status, hallucinations, hypertension, seizures, and acute visual changes. Imaging plays an important role in the diagnosis of this entity, especially by conventional and diffusion-weighted magnetic resonance imaging which enables an accurate diagnosis by identifying symmetric white matter lesions, especially in the parietal and occipital lobes. Herein, we report a 54-year-old female patient, newly diagnosed with non-metastatic moderately differentiated adenocarcinoma of the cecum. The patient received her first cancer chemotherapy (5-fluorouracil at 300 mg/m^2^). Five days later she was admitted to the intensive care unit for confusion following two generalized seizures. Conventional and diffusion-weighted magnetic resonance imaging was performed and showed diffuse white matter lesions of the parietal and occipital lobes. A diagnosis of 5-fluorouracil-induced leukoencephalopathy was established. The diagnosis of leukoencephalopathy should be considered in patients receiving cancer chemotherapy with alterations in mental status and seizures.

## Introduction

Chemotherapy-induced leukoencephalopathy is an uncommon complication of chemotherapy, characterized by specific clinical and neuroimaging findings [1]. At the clinical level, patients can present with neurological symptoms, such as seizures, visual impairment headaches, and altered mental functioning, frequently associated with arterial hypertension [1,2]. Known risk factors for this entity include chemotherapeutic agents such as fluoropyrimidines, immunosuppression, and renal dysfunction. However, the underlying mechanisms remain unclear [3]. Fluoropyrimidines are used to treat numerous types of cancer, including colon, ovarian, breast, gastric, and pancreatic cancers [4].

Imaging plays an important role in the diagnosis of this entity, especially by conventional and diffusion-weighted magnetic resonance imaging (MRI) which enables an accurate diagnosis by identifying symmetric white matter lesions, especially in the parietal and occipital lobes [5]. This condition has also been reported in children after cancer chemotherapy intake [6,7]. Herein, we report a case of 5-fluorouracil-induced toxic leukoencephalopathy in a 54-year-old female patient.

## Case presentation

In this study, we report a 54-year-old female patient, with no particular medical history. She was newly diagnosed with stage III B, non-metastatic moderately differentiated adenocarcinoma of the cecum. The patient received her first cancer chemotherapy by administration of 5-fluorouracil at 300 mg/m^2^. Five days later she was admitted to the intensive care unit for confusion following two new-onset generalized seizures.

At presentation, her vital signs were within normal limits except her blood pressure which was elevated at 167/103 mmHg. The mental status was altered with a Glasgow Coma Scale of 13. The heart rate was at 94 beats per minute, and the respiratory rate was at 15 breaths per minute. The body temperature was 37°C.

Neurologic examination revealed no abnormalities. The patient's cranial nerves, motor function, sensory function, and cerebellar function were all within normal limits. There were no signs of neurological deficits or underlying pathologies. Bilaterally positive Babinski sign was noted. Blood investigations including serum creatinine and urea, blood glucose, serum bilirubin, and alkaline phosphatase were normal. Cerebrospinal fluid examination showed no meningitis and no leptomeningeal involvement by neoplastic cells.

**Figure 1:**
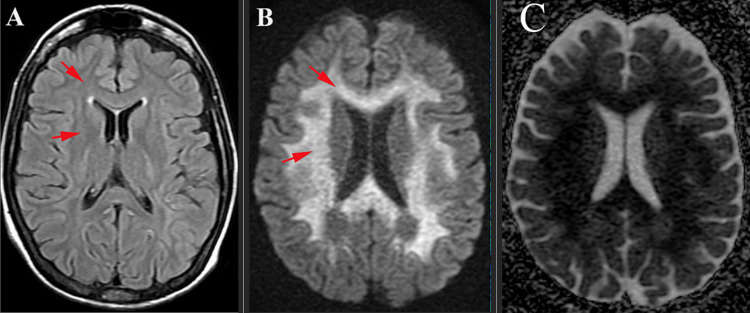
MRI of the patient performed approximately 5 hours post-seizure episodes. (A) Axial FLAIR sequence revealing hyperintensity of the white matter of the bilateral cerebral hemisphere in the frontoparietal region (red arrows), the corpus callosum, the internal capsules, and the cerebellar peduncles. (B) Diffusion-weighted magnetic resonance imaging demonstrates restricted diffusion on diffusion-weighted imaging (DWI) (red arrows). (C) Apparent diffusion coefficient (ADC) values were found to be 0.310-0.550 in affected white matter and 0.880 in unaffected white matter. FLAIR: fluid-attenuated inversion recovery

Magnetic resonance imaging was performed approximately 5 hours after the seizure episodes and identified T2 and fluid-attenuated inversion recovery (FLAIR) hyperintensity of the white matter of bilateral cerebral hemisphere in the frontoparietal region, the corpus callosum, the internal capsules, and the cerebellar peduncles (Figure 1, panel A). Diffusion-weighted imaging (DWI) sequence revealed diffusion restriction in the correlated areas as seen on T2-FLAIR sequence (Figure 1, panel B). Apparent diffusion coefficient (ADC) values were found to be 0.310 to 0.550 in affected white matter and 0.880 in unaffected white matter (Figure 1, panel C).

MRI with contrast injection has revealed no evidence of venous sinus thrombosis. A diagnosis of 5-fluorouracil-induced leukoencephalopathy was established. The decision to stop treatment with 5-fluorouracil and administration of an antiepileptic treatment was made. Follow-up of the patient showed full recovery of the patient after 13 days with no residual deficits. Control MRI revealed a complete reversal of the initial lesions, and chemotherapy could be resumed with oxaliplatin. The antiepileptic was ceased four months later. The patient will undergo repeat brain MRI every year.

## Discussion

Leukoencephalopathy is a rare, progressive condition characterized by damage to myelin and secondary structural damage to the brain’s white matter [4]. Many factors are known to induce or favor leukoencephalopathy and include infectious, metabolic, radiation, and toxic factors. Among toxic factors, cancer chemotherapy agents are known to induce potentially reversible leukoencephalopathy [8]. The most involved cancer chemotherapy agents include 5-fluorouracil (5-FU), methotrexate which is the most reported agent in the literature, vincristine, ifosfamide, cyclosporine, and cisplatin [8-10].

In our reported case, as well as in other types of malignancies like ovarian, stomach, and head and neck cancers, 5-FU is utilized as a treatment. This medication is a fluorine-substituted derivative of pyrimidine uracil and is specifically effective against solid tumors such as colon cancer. This agent acts by reducing the formation of thymidine monophosphate and incorporation into ribonucleic acid (RNA) and thus blocks desoxyribonucleic acid (DNA) synthesis [11]. The neurotoxicity secondary to 5-FU occurs with an incidence of 5% and is more frequent in cases of associated malnutrition and in female patients [11]. Some studies have also shown the presence of dihydropyrimidine dehydrogenase deficiency and hyperammonemia in cases of 5-FU-induced leukoencephalopathy [12,13]. In addition to leukoencephalopathy, fluoropyrimidines may induce many other adverse effects, such as GI complaints, bone marrow suppression, dermatitis, and alopecia [14].

Methotrexate, a cell cycle-specific, is a part of the therapeutic protocol for leukemias in children. It prevents the conversion of folic acid to tetrahydrofolic acid by inhibiting dihydrofolate reductase. Cases of methotrexate-induced leukoencephalopathy have been reported in both high-dose intravenous and intrathecal administrations, with an incidence ranging from 3% to 10% [15]. Risk factors for leukoencephalopathy in patients receiving therapy with methotrexate include a high dose, young age of patient, association with cranial irradiation, and route of methotrexate administration [15].

On the clinical level, a wide range of symptoms can be observed, such as dizziness, headache, depression, and, as reported in our case, confusion and seizures [16]. Other reported manifestations include ischemic attacks, cerebellar dysfunction, posterior reversible encephalopathy syndrome, and myopathy [16]. The clinical presentation is often considered to be heterogenous with the possible occurrence of severe presentation, such as coma and even death, especially if dose or regimen is not adjusted [14]. As in our case, patients receiving cancer chemotherapy with recent onset of neurologic symptoms should raise suspicion for chemotherapy-induced leukoencephalopathy. Distinction from stroke, metabolic disorders, infection, and other toxic leukoencephalopathies is crucial and can be sometimes challenging [14].

On the radiological level, magnetic resonance imaging (MRI) remains the best diagnostic modality. Conventional MRI reveals a diffuse T2 and FLAIR hyperintense signal in the white matter of the deep periventricular and corpus callosum. Basal ganglia, thalamus, and subcortical U-fibers are classically spared [17].

Diffusion-weighted MRI is an efficient technique in the diagnosis of leukoencephalopathy [17]. Diffusion-weighted MRI also helps in the follow-up of the affected patients after stopping the responsible treatment and shows improvement and resolution of diffusion restriction [18]. When performed, computed tomography (CT) scan reveals bilateral, symmetrical hypodense regions of the white matter [4,14]. Although imaging is useful for the positive diagnosis of leukoencephalopathy, it is not sensitive for determining its etiology.

No known specific treatment for cancer chemotherapy-induced leukoencephalopathy exists. Management of affected patients is based on the stoppage of the offending agent. Supportive corticosteroids and antioxidants may improve the clinical symptoms as well as the radiological lesions [4].

## Conclusions

Chemotherapy-induced leukoencephalopathy is an uncommon, but potentially severe complication of chemotherapy. As in our case, patients receiving cancer chemotherapy with recent onset of neurologic symptoms should raise suspicion for chemotherapy-induced leukoencephalopathy. Distinction from stroke, metabolic disorders, infection, and other toxic leukoencephalopathies is crucial and can be sometimes challenging. A wide range of symptoms can be observed, such as dizziness, headache, depression, and, as reported in our case, confusion and seizures with the possible occurrence of severe presentation, such as coma and even death. Although imaging is useful for the positive diagnosis of leukoencephalopathy, it is not sensitive for determining its etiology.
